# Bringing the Cognitive Estimation Task into the 21^st^ Century: Normative Data on Two New Parallel Forms

**DOI:** 10.1371/journal.pone.0092554

**Published:** 2014-03-26

**Authors:** Sarah E. MacPherson, Gabriela Peretti Wagner, Patrick Murphy, Marco Bozzali, Lisa Cipolotti, Tim Shallice

**Affiliations:** 1 Institute of Cognitive Neuroscience, University College London, London, United Kingdom; 2 Department of Neuropsychology, National Hospital for Neurology and Neurosurgery, London, United Kingdom; 3 Department of Psychology, Universidade Federal de Ciências da Saúde de Porto Alegre - UFCSPA, Porto Alegre, Brazil; 4 Wellcome Department of Imaging Neuroscience, University College London, London, United Kingdom; 5 Neuroimaging Laboratory, Santa Lucia Foundation, IRCCS, Rome, Italy; 6 Dipartimento di Psicologia, University of Palermo, Palermo, Italy; 7 Cognitive Neuroscience, International School for Advanced Studies (SISSA), Trieste, Italy; University of Bologna, Italy

## Abstract

The Cognitive Estimation Test (CET) is widely used by clinicians and researchers to assess the ability to produce reasonable cognitive estimates. Although several studies have published normative data for versions of the CET, many of the items are now outdated and parallel forms of the test do not exist to allow cognitive estimation abilities to be assessed on more than one occasion. In the present study, we devised two new 9-item parallel forms of the CET. These versions were administered to 184 healthy male and female participants aged 18–79 years with 9–22 years of education. Increasing age and years of education were found to be associated with successful CET performance as well as gender, intellect, naming, arithmetic and semantic memory abilities. To validate that the parallel forms of the CET were sensitive to frontal lobe damage, both versions were administered to 24 patients with frontal lobe lesions and 48 age-, gender- and education-matched controls. The frontal patients’ error scores were significantly higher than the healthy controls on both versions of the task. This study provides normative data for parallel forms of the CET for adults which are also suitable for assessing frontal lobe dysfunction on more than one occasion without practice effects.

## Introduction

In everyday life, cognitive estimation is an important form of problem solving. Given that previously learned knowledge cannot be directly called upon, so that the exact answer is not known, to reach an appropriate answer requires the development of an appropriate strategy and reasoning (e.g., estimating how much your grocery shopping will cost). To produce reasonable cognitive estimates, individuals need to identify and select the appropriate cognitive set, retrieve and manipulate particular details or estimates from that cognitive set, monitor the appropriateness of their response and repeat the procedure if necessary to produce a better estimate.

The Cognitive Estimation Task (CET) was devised by Shallice and Evans [1] in an attempt to assess the ability to provide appropriate cognitive estimates. Shallice and Evans [1] found that patients with damage to the frontal lobes performed poorly on the task producing bizarre over- or under-estimates. Many of the cognitive abilities thought to be important for producing successful cognitive estimates are executive in nature. Executive functions are thought to be mediated mainly by the frontal lobes [2]. Thus, it is not surprising that frontal lobe damage produces deficits in estimation. Since this original study, a number of researchers have demonstrated deficits in cognitive estimation in patients with frontal lobe lesions compared to patients with temporal or diencephalic lesions and healthy controls [3]–[6]. However, it should be noted that Taylor and O’Carroll [7] did not find a significant difference between patients with anterior and posterior lesions in terms of cognitive estimation in a large group of patients with different neurological conditions. Deficits in CET performance have also been reported in Alzheimer’s disease [6]–[13], Korsakoff’s disease [11], [14], [15], frontotemporal dementia [12], subcortical vascular dementia [16], post-encephalitis amnesia [17], major depressive disorder [18], traumatic brain injury [19] and in some cases of non-demented Parkinson’s disease [20] but see [21].

The CET devised by Shallice and Evans [1] included 15 questions. The possible answers were coded according to the degree of bizarreness using a 4-point system. The CET was administered to a group of 45 frontal patients, a group of 51 posterior patients and a control group of twenty-five patients with extra-cerebral lesions. The results revealed that the percentage of very extreme, extreme, and quite extreme responses produced by the frontal patients was significantly greater than the control group, indicating a frontal lobe deficit associated with poor performance on the task.

The CET became a relatively widely used test of executive functions [22]. However, a number of issues have been raised concerning both the original version of the CET, as well as versions developed subsequently. For example, in the original version of the CET, there was only a small control group and there was no published normative data [1], [10], [13], [23]. It could also be argued that certain items are only answerable by individuals from the specific country the normative data were obtained [13], [24]–[27]. Axelrod and Millis [24] produced normative data for a revised version of the CET based on a larger group of 164 healthy American volunteers. Around the same time, British normative data for a shortened version of the original CET [23] was collected by O’Carroll, Egan, and MacKenzie [25] from a sample of 150 participants. Furthermore, normative data have been obtained from individuals with limited education ranges. For instance, Axelrod and Millis [24] recruited more highly educated individuals whereas O’Carroll et al. [25] focused more on participants with fewer years of education. Other studies have collected normative data for versions of the CET which include items thought to rely less on general knowledge [28] and items for use with individuals from different cultures [13], [26], [27] but many of these include items that are now outdated [1], [23], [25]. For a review of the different CET versions used in healthy and clinical populations see Wagner, MacPherson, Parente and Trentini [29].

Repeated assessments of ‘executive’ functions are often required to monitor a large number of neurological conditions. Therefore, to have multiple versions of an executive task is an undoubted advantage. In the specific case of the CET, the questions asked should be novel, if administered on several occasions. This would avoid the possibility to have subjects thinking about possible responses after the test or remembering answers given previously. Also, to the best of our knowledge parallel versions of the CET do not exist. The aim of this study is to devise two parallel standardized versions of the CET which contain more up-to-date landmarks, people and objects that everyone will be familiar with. Normative data on a large number of healthy controls varying in age, gender and years of education will be provided. Finally, the performance of frontal patients on these parallel forms will be assessed.

## Methods and Materials

### Experiment 1 Participants

One hundred and eight-four healthy British volunteers (81 men, 103 women) aged between 18 and 79 years (M = 48.07 years, SD = 17.51 years) were recruited for the study. Their level of education ranged from 9–22 years (M = 14.33 years, SD = 2.92 years). Participants were grouped into different decades according to their ages: 18–29 years, 30–39 years, 40–49 years, 50–59 years, 60–69 years and 70–79 years, as well as in relation to their different levels of education in the UK: 9–11 years (O level or Standard Grade examinations), 12–15 years (A-level or Higher Grades examinations as well as College level higher education) and 16 plus years (University level education). One hundred and sixty-nine participants were right handed. None of them had any previous history of head injury or stroke, major neurological or psychiatric illness, or alcohol abuse as listed in the exclusion criteria for the Wechsler Adult Intelligence Scale-III UK (WAIS-III UK) [30] and the Wechsler Memory Scale-III (WMS-III UK) [31]. The majority of participants were recruited through the Institute of Cognitive Neuroscience, University College London volunteer panel and the Department of Psychology, University of Edinburgh volunteer panel. Others were recruited through an advertisement in a local newspaper, through personal contact with the researchers or word-of-mouth. Participants were reimbursed for any expenses for their participation. All participants spoke English as their first language. The study was approved by the National Hospital for Neurology and Neurosurgery & Institute of Neurology Joint Research Ethics Committee in London and the Philosophy, Psychology and Language Sciences Research Ethics Committee in Edinburgh. Written consent was obtained according to the Declaration of Helsinki. The demographic information of the participants according to age, education and gender are shown in Table 1.

**Table 1:** Distribution of the participants’ demographic characteristics according to age, education and gender.

		Age (years)						
		18–29	30–39	40–49	50–59	60–69	70–79	Total
Education (years)								
9–11	M	2	3	5	3	6	2	21
	F	5	0	3	3	6	5	22
12–15	M	9	4	3	6	6	4	32
	F	5	4	7	7	5	5	33
16–22	M	7	5	5	5	3	3	28
	F	8	11	6	12	6	5	48
Total	M	18	12	13	14	15	9	81
	F	18	15	16	22	17	15	103

M = Male; F = Female.

### Experiment 1 Background Neuropsychological Measures

All participants performed the National Adult Reading Test-Revised (NART) to estimate verbal intelligence [32] and Raven’s Advanced Progressive Matrices to assess nonverbal abstract reasoning (APM) [33]. The Graded Naming Test (GNT) [34] was administered to assess naming abilities and the Graded Difficulty Arithmetic (GDA) [35] to assess arithmetical abilities. The Information subtest from the WAIS [30] was administered to assess general knowledge.

### Experiment 1 Cognitive Estimation Task (CET)

Fifty-eight estimation questions were devised by the authors who included items relating to length (12), weight (10), area (10), speed (10) and number (16). All items required numerical responses. Participants were told that for most questions there was no exact answer or it was unlikely they would know the answer so they should make a reasonable guess or estimate of what the answer would be. The estimation questions were asked out loud by the experimenter and participants gave their answers orally. Participants could answer the items using their preferred unit of measurement, but when scoring the items, the responses were converted to the same unit of measurement. This was to ensure that participants did not fail to provide an appropriate estimate due to unfamiliarity with the unit of measurement rather than poor estimation abilities. Individuals were given as much time as necessary to produce estimations. For each item, participants were asked if they were sure that the response they had provided was a reasonable estimate and, if not, they were able to change their response.

### Experiment 1 Data Analysis

Internal consistency of the CET items was examined using Cronbach’s alpha coefficient [36] and Guttman split-half reliability coefficient [37]. Spearman’s correlation coefficients were calculated to examine the relationship between the participants’ CET performance and the background neuropsychological measures as well as age, gender and education. Linear regression analyses were conducted to determine which descriptive characteristics and neuropsychological measures are significant predictors of cognitive estimation. Finally, principal component analyses (PCA) were conducted on each 9-item CET with orthogonal rotation (varimax) to determine the number of components each CET loads upon.

### Experiment 2 Participants

Twenty-four patients (14 men and 10 women) with lesions localised within the frontal lobes were recruited from the National Hospital for Neurology and Neurosurgery. Inclusion and exclusion criteria were: (1) the presence of a focal lesion confined to the frontal lobes based on a clinical CT or MRI scan, (2) English as a first language, (3) absence of childhood onset epilepsy (late onset seizures arising from the lesion were allowed), (4) absence of severe aphasia, and (5) absence of other significant neurological and psychiatric disorders. The aetiologies were as follows: glioma = 12, meningioma = 6, subarachnoid haemorrhage (SAH) = 3, space occupying lesion (SOL) = 1, anterior communicating artery aneurysm (ACoAA) = 1 and arteriovenus malformation (AVM) = 1. Twelve of the 18 tumour patients had undergone surgical excisions. Three of the 6 remaining tumour patients had undergone CT stereotaxic biopsies without excision. Frontal lesions were localised by operation site in the case of surgical patients or by gross tumour characterisation in the non-surgical glioma, SOL, SAH, ACoAA and AVM patient groups. Lesion characterisation was on the basis of clinical MRI scans (or CT scans where MRI was unavailable). The mean time since surgery (excluding the 3 nonsurgical glioma patients) was 11.64 months (SD = 16.61 months, range = 1–50 months). Five patients were left handed (2 with left hemisphere lesions, 2 with right hemisphere lesions and 1 with a bilateral lesion). The mean age of the patient group was 47.96 years (SD = 13.11 years, range = 27–73 years) and the mean education was 14.83 years (SD = 3.20 years, range = 10–19 years).

Forty-eight healthy volunteers (28 men and 20 women) from Experiment 1 were selected as controls for the 24 frontal patients. Two healthy volunteers were selected for each patient, both matched in terms of gender as well as age and years of full-time education (plus or minus a maximum of 3 years). The mean age of the healthy control group was 47.92 years (SD = 13.11 years, range = 25–75 years) and the mean education was 14.67 years (SD = 3.26 years, range = 10–21 years). An independent samples t-test demonstrated that the frontal patients and the control volunteers did not differ significantly in terms of age (p = .99) or years of education (p = .84). Consent was obtained according to the Declaration of Helsinki and the study was approved by the National Hospital for Neurology and Neurosurgery & Institute of Neurology Joint Research Ethics Committee.

## Results

### Experiment 1 Background Neuropsychological Measures

Table 2 demonstrates the means and standard deviations for the volunteers performing the background neuropsychological measures.

**Table 2:** Performance of the 184 healthy volunteers on the background neuropsychological measures.

	Mean	Standard Deviation	Minimum	Maximum
NART IQ	114.51	9.41	84	131
Raven’s APM (max = 12)	8.84	2.10	1	12
GNT (max = 30)	23.49	4.02	11	30
GDA (max = 24)	16.18	4.80	4	26
Information Subtest (max = 29)	22.64	4.16	8	29

NART = National Adult Reading Test; APM = Advanced Progressive Matrices; GNT = Graded Naming Test; GDA = Graded Difficulty Arithmetic.

### Experiment 1 Cognitive Estimation Task (CET)

Firstly, any items that were predominantly British or resulted in ambiguity were removed. The means and standard deviations for the remaining items were then examined. Items where the standard deviation was greater than the mean were removed to reduce the items with the greatest variance in terms of healthy individuals’ responses. This resulted in 38 estimation items. The percentiles for each item’s actual responses were then examined and outliers that were 1.5 or more times the interquartile range were removed. Responses between the 20^th^ and 80^th^ percentile were considered normal and awarded 0 points. Responses that were equal to or more than the 10^th^ but less than the 20^th^ percentile or more than the 80^th^ percentile but less than or equal to the 90^th^ percentile were considered quite extreme and awarded 1 point. Responses were considered extreme when they were more than or equal to the 5^th^ percentile but less than the 10^th^ percentile or more than the 90^th^ percentile but less than or equal to the 95^th^ percentile and scored 2 points. Finally, responses less than the 5^th^ or more than the 95^th^ percentile were considered very extreme and scored 3 points. Any missing values that were due to the removal of outliers or where participants were unable to provide an estimate were scored as very extreme and awarded 3 points. The descriptive statistics for the 184 healthy participants’ actual responses to the 38 CET items when outliers were removed and their percentile ranges are in the supporting information file (Tables S1 and S2).

The frequency distributions for the number of times scores of 0, 1, 2 and 3 occurred for each item were then examined. According to the normal distribution, only 5% of scores at each end of the curve should be scored as extreme values (i.e., 3 points). Items are more likely to be useful for detecting abnormality if there are relatively few outliers in the normal population and when normal subjects are more consistent in the responses chosen. The number of times 3 would be allocated for an item would be increased with the number of outliers and the degree to which the more extreme values found were less consistently chosen in the normal population. Therefore, any items where 20 or more individuals achieved a score of 3 were excluded. Participants could then obtain a total score between 0 and 72 for the 24 CET items where the higher the score, the greater the number of responses that deviated from the group. The mean error score for the CET for the entire sample was 14.04 out of a possible 72 (SD = 7.21, range = 1–56).

The Cronbach’s alpha for the 24 items on the CET was.62, which is considered an acceptable level of internal reliability [38], [39]. To assess whether the scores for each CET item correlated with the scores for the other CET items, item-total correlations between the individual item score and the total score for the remaining items were also conducted (see Table S3 in the supporting information file for individual items). Those items that were not significant were removed, resulting in 19 CET items and the one remaining item relating to the area dimension was also removed. The remaining 18 CET items had a Cronbach’s alpha of.63 and the Guttman split-half reliability coefficient was.59.

Spearman’s correlational analyses were then conducted to investigate whether performance on the 18-item CET correlates with performance on the background neuropsychological measures. Table 3 demonstrates the correlational analyses for CET performance and the background neuropsychological measures. All measures significantly negatively correlated with CET performance. This suggests that the better the performance on the background measures (i.e., NART IQ, Raven’s APM, GNT, GDA and Information subtest), the lower the error score on the CET. The background measures were then entered into a linear regression analysis to investigate their involvement in CET performance. The analysis revealed a statistically significant model that explains 33.9% of the variance on the CET where performance on the GDA (*p*<.05) and the Information subtest significantly influences performance (*p*<.0001).

**Table 3:** Correlational analyses between CET performance and the background neuropsychological measures.

	CET Performance	
	r	p-value
NART IQ	−.41	<.0001
Raven’s APM	−.30	<.0001
GNT	−.41	<.0001
GDA	−.39	<.0001
Information Subtest	−.56	<.0001

NART = National Adult Reading Test; APM = Advanced Progressive Matrices; GNT = Graded Naming Test; GDA = Graded Difficulty Arithmetic.

The 18-item CET was then subdivided into two 9-item parallel forms of the CET with a total error score ranging between 0 and 27 (Version A: M = 5.02, Mdn = 4.00, SD = 3.51, range = 0–24; Version B: M = 5.42, Mdn = 5.00, SD = 3.89, range = 0–22). The means, standard deviations, and the minimum and maximum values for the actual responses provided for each CET version are shown in Tables 4 and 5. When the 18-items were split into two 9-item versions, both the CET A and CET B had relatively low reliability, Cronbach’s α = .44 and.51 respectively. The Guttman split-half reliability coefficients were.47 and.59 respectively.

**Table 4:** The means, standard deviations, minimum and maximum values and principal component analysis for version A of the CET.

								Rotated Components		
	Category	Unit	Mean	SD	Min	Max	1st	PC1	PC2	PC3
What is the maximum speed of a Harley-Davidson motorbike?	Speed	km/h	237.83	64.80	100.00	483.00	**.46**	.19	.15	**.64**
What is the length of the average newborn baby?	Length	cm	42.71	13.16	11.00	80.00	**.45**	-.02	**.66**	.14
How fast do race horses run?	Speed	km/h	56.62	23.07	0.81	128.75	**.49**	**.67**	.05	-.01
What is the average jogging speed?	Speed	km/h	9.78	4.10	0.81	20.92	**.57**	**.56**	**.31**	-.03
How many segments are there in an orange?	Quantity	segments	12.20	3.99	4.00	30.00	**.46**	**.33**	**.30**	.12
What is the length of a new pencil?	Length	cm	17.62	4.66	6.00	35.00	**.37**	.04	**.69**	-.24
What is the maximum speed of a cheetah?	Speed	km/h	118.04	61.11	10.00	435.00	**.37**	**.69**	-.25	.13
What is the length of an average men's mountain bike?	Length	m	1.56	0.45	0.30	3.66	**.43**	.09	**.44**	.26
How many keys are there on a standard computer keyboard?	Quantity	keys	51.56	15.17	25.00	126.00	.21	-.08	-.03	**.77**
Eigenvalue							1.69	1.41	1.38	1.18
Variance (%)							18.78	15.61	15.38	13.11

Loadings >.30 in bold; 1^st^ = 1^st^ unrotated.

**Table 5:** The means, standard deviations, minimum and maximum values and principal component analysis for version B of the CET.

								Rotated Components			
	Category	Unit	Mean	SD	Min	Max	1^st^	PC1	PC2	PC3	PC4
What is the average walking speed of the typical healthy adult man?	Speed	km/h	6.11	4.09	0.81	40.23	**.43**	.19	-.18	**.67**	**.46**
How long is the average tie?	Length	m	0.86	0.35	0.11	2.74	.25	.02	**.46**	-.05	.07
What is the fastest tennis serve?	Speed	km/h	166.62	74.62	2.00	483.00	**.64**	**.60**	**.33**	.15	-.09
How many keys are there on a standard piano?	Quantity	keys	66.18	32.35	4.00	200.00	**.52**	.12	**.62**	.07	**.33**
What is the age of the oldest person in your country?	Quantity	age	116.00	6.76	102.00	140.00	**.42**	**.64**	-.16	.02	.24
What is the length of an average man's spine?	Length	cm	87.90	22.26	18.00	183.00	**.43**	.06	**.68**	.13	-.15
What is the maximum speed of a cyclist?	Speed	km/h	57.85	32.40	6.00	241.00	.22	-.04	.16	-.04	**.85**
How many strings are there on a harp?	Quantity	strings	41.22	34.05	4.00	267.00	**.44**	.01	.19	**.84**	-.18
What is the maximum speed of a Formula 1 car?	Speed	km/h	346.88	89.73	84.00	805.00	**.60**	**.79**	.15	.01	-.11
Eigenvalue							1.89	1.45	1.31	1.20	1.18
Variance (%)							20.97	16.15	14.58	13.32	13.12

Loadings >.30 in bold; 1^st^ = 1^st^ unrotated.

According to Kaiser-Meyer-Olkin (KMO), the sample for version A of the CET were adequate for PCA, KMO = .57, with individual item KMO values >.50. The correlations between items were also sufficiently large for PCA with Bartlett’s test of sphericity, χ^2^ (36) = 60.22, p<.01, indicated that correlations between items were adequate for PCA. The analysis revealed that 3 factors could be extracted from the data, each with an eigenvalue greater than 1 and explaining 44.09% of the variance (see Table 4).

For the 9-item CET B, the sample were also suitable for PCA, KMO = .61, with KMO values >.49 for individual items. Bartlett’s test of sphericity indicated the correlations between items were large enough for PCA, χ^2^ (36) = 90.307, p<.0001. PCA revealed a four factor solution, each factor with an eigenvalue more than 1, and explaining 57.17% of the variance (see Table 5). If the item, “What is the maximum speed of a cyclist?” was removed from the analysis due to the lower KMO value of.49, the PCA revealed a three factor solution, each factor with an eigenvalue over 1, explaining 49.92% of the variance.

The means and standard deviations for 184 participants performing versions A and B of the CET respectively according to age group, gender and level of education are shown in the supporting information file (Tables S4 and S5). Spearman’s rank order correlations revealed that performance on the two versions of the CET correlated significantly (r = .34, p<.0001).

Spearman’s rank order correlations were calculated to examine whether performance on the two versions of the CET correlates with age or education. The performance on the CET was significantly negatively correlated with age (version A: r = -.16, p<.05; version B: r = -.17 p<.05) suggesting that the older the individual, the better the performance on the CET. There were also significant negative correlations between CET performance on version A and version B and education (version A: r = −0.20, p<.01; version B: r = −0.21, p<.01), suggesting the higher the level of education, the better the CET performance. Separate Mann-Whitney U-Tests revealed a significant main effect of gender on both versions of the CET [version A: U = 5199.50, z = 2.88, p<.005; version B: U = 5484.00, z = 3.68, p<.0001] where male participants (version A: M = 4.22, SD = 3.16; version B: M = 4.15, SD = 2.99) produced significantly lower CET scores than female participants (version A: M = 5.65, SD = 3.65; version B: M = 6.42, SD = 4.22).

Separate linear regression analyses were conducted to investigate the involvement of age, gender and years of education on versions A and B of the CET. The analysis of version A revealed a statistically significant model that explains 10.1% of the variance on the CET where age, gender and education all significantly influence performance (p≤.05). The model for version B explains 18.4% of the variance on the CET where age (p<.01), gender (p<.0001) and education all influence performance (p<.0001). The correction grids to obtain adjusted scores for the different age, education and gender combinations for versions A and B of the CET, as well as the corrections for the combinations not reported, are in Tables S6 and S7 in the supporting information file.

The distribution of the error scores for version A of the CET adjusted for age, gender and education is as follows: M = 5.93, Mdn = 5.50, SD = 4.09, minimum = −3, maximum = 27, interquartile range = 6. The distribution of the CET scores for version B adjusted for age, gender and education is: M = 6.84, Mdn = 6.00, SD = 4.90 minimum = −2, maximum = 26, interquartile range = 5. Table 6 provides the percentiles of the distribution of the CET scores adjusted for age, gender and education.

**Table 6:** Percentiles of the distribution of the adjusted error scores on the parallel versions of the CET.

Percentiles	CET A	CET B	Percentiles	CET A	CET B
5^th^	0	0	55^th^	6	6
10^th^	1	1	60^th^	7	7
15^th^	2	2	65^th^	7	8
20^th^	2	3	70^th^	8	9
25^th^	3	4	75^th^	9	9
30^th^	4	4	80^th^	9	10
35^th^	4	5	85^th^	10	12
40^th^	4	5	90^th^	11	14
45^th^	5	6	95^th^	13	16
50^th^	6	6	100^th^	27	26

The possible scores range from zero (best performance) to 27 (worst performance).

### Experiment 2 Background Neuropsychological Measures

Each frontal patient performed the same background neuropsychological assessment as the healthy participants in Experiment 1.

### Experiment 2 Cognitive Estimation Task (CET)

The frontal patients were then administered both versions A and B of the final 9-item CET using the same instructions as Experiment 1.

### Experiment 2 Data Analysis

The performance of the frontal patients and healthy controls was compared using two-tailed independent samples *t*-tests when the data were normally distributed and Mann-Whitney U-Tests when the data were not normally distributed.

## Results

### Experiment 1 Background Neuropsychological Measures

Table 7 shows the means and standard deviations for the frontal patients and healthy control groups performing the background measures. A Mann-Whitney U-Test revealed that the frontal patients correctly answered significantly fewer arithmetical problems than the healthy controls (U = 384.00, z = −2.30, p<.05). However, the frontal group did not significantly differ from the control group on any of the other background measures (p>.10).

**Table 7:** Performance of the frontal and healthy control groups on the background measures and the parallel forms of the CET.

	NART IQ	Raven's APM (max = 12)	GNT (max = 30)	GDA (max = 24)	Information (max = 29)	CET A (max = 27)	CET B (max = 27)
Frontal Patients	112.39	8.87	22.09	13.25	22.04	8.63	7.21
	(8.34)	(2.87)	(4.56)	(5.64)	(4.77)	(5.40)	(3.88)
Frontal Controls	115.27	9.04	23.98	16.46	23.08	5.38	5.17
	(7.28)	(2.06)	(3.28)	(4.24)	(3.83)	(3.35)	(3.55)

NART = National Adult Reading Test; APM = Advanced Progressive Matrices; GNT = Graded Naming Test; GDA = Graded Difficulty Arithmetic.

### Experiment 2 Cognitive Estimation Task (CET)

The means for the patient groups and healthy controls performing versions A and B of the CET are also in Table 7. Mann-Whitney U-Tests revealed the frontal patients performed significantly more poorly than the healthy controls on both versions of the CET, achieving higher error scores than the control group (version A: U = 788.00, z = 2.54, p<.05 and version B: U = 753.50, z = 2.13, p<.05). Spearman’s rank order correlational analysis revealed that the performance of the frontal patients on the two versions of the CET was significantly correlated (r = .45 p<.05). However, unlike our healthy participants, the frontal patients’ CET performance did not correlate significantly with age (p>.12).

To determine whether the CET is equally sensitive with both male and female frontal patients, further analysis revealed that the male frontal patients performed significantly more poorly than the healthy controls on both version A (frontal patients: M = 8.29, SD = 5.80; controls: M = 5.00, SD = 3.54, *t*(40) = −2.28, p<.05) and version B (frontal patients: M = 6.71, SD = 2.53; controls: M = 4.18, SD = 2.97, U = 291.00, z = 2.56, p<.05) of the CET. In the female participants, the frontal patients performed significantly more poorly than the healthy controls on version A (frontal patients: M = 9.10, SD = 5.07; controls: M = 5.90, SD = 3.08, *t*(28) = −2.16, p<.05) but not version B (frontal patients: M = 7.90, SD = 5.32; controls: M = 6.55, SD = 3.89, p = .68).

## Discussion

This study provides normative data for two newly devised versions of the CET. Attempts have been made to include up-to-date concepts which are no longer specific to certain countries such as UK or USA. Age, gender and education were all found to be associated with successful CET performance in healthy individuals. CET performance was also negatively correlated with intellect, naming, arithmetic and semantic memory abilities. Frontal patients were also found to produce significantly higher CET error scores than age, gender and education matched controls. This would suggest that our parallel versions of the CET are suitable for assessing frontal lobe dysfunction in clinical practice and research on more than one occasion to the same individual without practice effects.

The original version of the CET did not explicitly provide participants with the opportunity to change their response if they felt it was inappropriate, although changes to the estimates were accepted. Poor performance on the CET in frontal patients may be due to their impulsive nature where they simply respond with the first answer that comes to mind without monitoring the appropriateness of the response. In the new versions of the CET, participants were encouraged to review their responses in order to examine whether this would result in better estimations. However, our data suggest that this is not the case and even when frontal patients are encouraged to evaluate their responses and change them if necessary, they produce bizarre estimations. Further work is required to determine whether frontal patients are disadvantaged by the inclusion of this extra step on the CET due to a lack of insight into their own estimation abilities.

The negative correlation with age and CET performance in our normative sample suggests that the older the participant, the better the performance on the task. It may be that our older individuals’ better performance on the CET is due to their ability to compensate for poorer reasoning and self-monitoring through intact semantic or factual knowledge. For example, to provide a fitting estimate for the item, “How many strings are there on a harp?”, one needs to access semantic knowledge about musical instruments. Indeed, both the current and previous findings in the literature have reported significant associations between CET performance and semantic knowledge [6], [11], [14], [25].

Improved CET performance with age would not be a reason to conclude that the CET is not an appropriate measure of executive dysfunction. For example, there are a large number of studies that have shown that phonemic fluency, another measure of executive dysfunction, does not decline with age or may in fact improve with age [40]–[43]. Moreover, while our older adults may have compensated for poorer reasoning and self-monitoring through intact semantic or factual knowledge, this could not explain the poor performance of our frontal group. Indeed, significant correlations between age and CET performance in our frontal patients were not found. Moreover, our frontal patients did not significantly differ from the controls in terms of their NART score or general knowledge performance, and yet they still produced significantly higher CET error scores. A plausible account is that performance on the CET requires both adequate strategic processes and good general knowledge. Therefore, while good general knowledge is necessary to perform well on the CET, without adequate strategic processes it is not sufficient to score within the normal range. In contrast, semantic retrieval on the NART and other neuropsychological tests of general knowledge does not require executive control.

An advantage of the current study was the inclusion of a large number of participants whose levels of education varied greatly. Previous attempts to provide normative data for the CET have tended to include individuals with either high or low education levels [24], [25] or at least have not specified what the participants’ education levels are [28]. The current findings support earlier studies that have found that the higher the number of years of education, the better the performance on the CET [24], [25], [27]. Significant correlations were also found between CET performance and intellectual abilities as has been shown with earlier versions of the CET correlating with novel abstract reasoning [11] and general intellectual abilities assessed using the WAIS [23] and the Wechsler Intelligence Scale for Children (WISC-III) [44]. It may be that some of the variance on the CET is due to education or general intellectual abilities rather than estimation abilities.

Few studies in the literature have reported whether gender influences CET performance. However, our CET advantage in male participants has previously been reported by other CET studies in the literature [25], [27], [45]. Moreover, while typically executive measures do not show gender differences, normative data for the Controlled Oral Word Association Test, one of the most widely used tests of frontal executive dysfunction, has provided evidence of gender effects where women perform better than men on the FAS phonemic fluency task [46], [47].

Therefore, while gender effects on executive tasks are not common, they would not speak against the claim that CET performance is a sensitive measure of executive abilities. As the Information subtest of the WAIS [30] and the Grading Difficulty Arithmetic Test (GDA) [35] were significant predictors of CET performance, higher estimation error scores in females might be explained in terms of poorer semantic knowledge and arithmetical abilities. Further analysis revealed that both versions of the CET were sensitive to frontal lesions in male patients but only version A in female frontal patients. However, there was a trend for the female frontal patients to also perform more poorly than healthy controls on version B. It may be that this lack of sensitivity in female patients is due to the small sample size. Further work is required to determine whether this is the case.

Clinicians and researchers generally use the CET to assess executive dysfunction [45]. However, our correlational analyses have shown that cognitive abilities such as abstract reasoning, general knowledge, naming and arithmetical abilities may also underlie the computation and evaluation of reasonable cognitive estimates. The CET appears to be multidimensional in nature with different cognitive functions operating in concert to achieve the overall goal. These findings also suggest that impairments in distinct cognitive abilities might underlie the CET impairments reported in individuals with syndromes such as Alzheimer’s disease [6]–[13], Korsakoff’s disease [11], [14], [15], frontotemporal dementia [12], subcortical vascular dementia [16] and post-encephalitis amnesia [17].

One limitation of this study is the lack of the inclusion of a posterior control group. If the CET is indeed sensitive specifically to frontal lobe lesions, one would predict that patients with non-frontal lesions should perform similarly to healthy controls when asked to produce cognitive estimates. Indeed, posterior patients produce significantly less extreme responses than frontal patients on the original version of the CET [1], [5]. Future work should attempt to demonstrate that patients with non-frontal lesions are able to produce appropriate cognitive estimates on these new versions of the CET, in order to provide evidence on the frontal lobe localisation of CET processes.

In terms of test reliability, the internal consistency of the items when they were split into versions A and B of the CET was low [39], suggesting that the test items within the same version of the CET vary and are not necessarily measuring the same construct. Moreover, PCA revealed that the items in the CET loaded on several different components, which supports the multidimensional nature of the task as well as executive abilities in general [48]. It also should be noted, however, that the value of Cronbach’s alpha can be deflated when there are a small number of items and multiple dimensions [49]. Previous versions of the CET have also been shown to have low reliability, as well as correlating with several cognitive domains and loading on more than one component [25], [45]. The advantages of the CET as a clinical tool include its ability to be administered quickly, at a bedside and without making any motor demands [45]. The CET also has the advantage of not demonstrating significant declines in performance as a result of healthy adult ageing and therefore might be useful to accurately classify individuals in terms of normal and abnormal ageing. Of course, this is only the initial stage in establishing the usefulness of the new parallel versions of the CET in clinical assessment and further research is required to examine whether performance the task can be localised to a specific subregion of the frontal lobes and the predictive accuracy of the test in terms of different forms of dementia.

## Supporting Information

Table S1 **The means, standard deviations, minimum and maximum values for the 38 items on the Cognitive Estimation Task.**
(DOCX)

Table S2 **Percentiles for individual items on the CET.**
(DOCX)

Table S3 **Internal consistency for the 24 CET items.**
(DOCX)

Table S4 **Means with standard deviations in parentheses per age, gender and education group for 184 participants performing version A of the CET.**
(DOCX)

Table S5 **Means with standard deviations in parentheses per age, gender and education group for 184 participants performing version B of the CET.**
(DOCX)

Table S6 **The correction grid with the points to add or subtract from the raw scores to obtain adjusted scores for version A of the CET.** For the combinations not reported, the corrections that should be applied to the raw CET scores to achieve adjusted scores are below the table.
(DOCX)

Table S7 **The correction grid with the points to add or subtract from the raw scores to obtain adjusted scores for version B of the CET.** For the combinations not reported, the corrections that should be applied to the raw CET scores to achieve adjusted scores are below the table.
(DOCX)
