# Correction: Bringing the Cognitive Estimation Task into the 21^st^ Century: Normative Data on Two New Parallel Forms

**DOI:** 10.1371/journal.pone.0104483

**Published:** 2014-07-29

**Authors:** The PLOS ONE Staff

There are errors in Table 6, the last paragraph of the Experiment 1 Cognitive Estimation Task (CET) section, and Tables S6 and S7. Please view the correct paragraph and tables here:

The distribution of the error scores for version A of the CET adjusted for age, gender and education is as follows: M  =  4.11, Mdn  =  4.00, SD  =  3.32, minimum  =  -2, maximum  =  21, interquartile range  =  4.00. The distribution of the CET scores for version B adjusted for age, gender and education is: M  =  3.99, Mdn  =  4.00, SD  =  3.50, minimum  =  -3, maximum  =  18, interquartile range  =  4.00. Table 6 provides the percentiles of the distribution of the CET scores adjusted for age, gender and education.

**Table 6:** Percentiles of the distribution of the adjusted error scores on the parallel versions of the CET. The possible scores range from zero (best performance) to 27 (worst performance).

Percentiles	CET A	CET B	Percentiles	CET A	CET B
5^th^	-1	-1	55^th^	4	4
10^th^	0	0	60^th^	5	5
15^th^	1	1	65^th^	5	5
20^th^	1	1	70^th^	6	5
25^th^	2	2	75^th^	6	6
30^th^	2	2	80^th^	7	7
35^th^	3	2	85^th^	7	8
40^th^	3	3	90^th^	8	9
45^th^	3	3	95^th^	10	10
50^th^	4	4	100^th^	21	18

## Supporting Information

Table S6 **The correction grid with the points to add or subtract from the raw scores to obtain adjusted scores for version A of the CET.** For the combinations not reported, the corrections that should be applied to the raw CET scores to achieve adjusted scores are below the table.
(DOCX)

Table S7 **The correction grid with the points to add or subtract from the raw scores to obtain adjusted scores for version B of the CET.** For the combinations not reported, the corrections that should be applied to the raw CET scores to achieve adjusted scores are below the table.
(DOCX)
