# Supplementary material for: Bringing the Cognitive Estimation Task into the 21st Century: Normative Data on Two New Parallel Forms
Source: PLoS One. 2014 Mar 26;9(3):e92554. doi: 10.1371/journal.pone.0092554 (PMC3966793; doi:10.1371/journal.pone.0092554)
Supplement: Table S1 — The means, standard deviations, minimum and maximum values for the 38 items on the Cognitive Estimation Task. (DOCX) [file pone.0092554.s001.docx]

|  | Item | Category | Unit | Mean | SD | Minimum | Maximum |
| --- | --- | --- | --- | --- | --- | --- | --- |
| 1. | What is the area (length and breadth) of the smallest postage stamp in your country?* | Area | cm | 3.68 | 2.36 | 0.20 | 10.00 |
| 2. | What is the maximum speed of a Harley-Davidson motorbike? | Speed | km/h | 237.83 | 64.80 | 100.00 | 483.00 |
| 3. | What is the area (length and breadth) of a single bed sheet?* | Area | m^2^ | 2.74 | 1.54 | 0.03 | 9.00 |
| 4. | What is the length of the average newborn baby? | Length | cm | 42.71 | 13.16 | 11.00 | 80.00 |
| 5. | How fast do race horses run? | Speed | km/h | 56.62 | 23.07 | 0.81 | 128.75 |
| 6. | What is the average jogging speed? | Speed | km/h | 9.78 | 4.10 | 0.81 | 20.92 |
| 7. | How many segments are there in an orange? | Quantity | segments | 12.20 | 3.99 | 4.00 | 30.00 |
| 8. | What is the length of a new pencil? | Length | cm | 17.62 | 4.66 | 6.00 | 35.00 |
| 9. | How heavy is a pair of men’s trainers? ^#^ | Weight | kg | 1.20 | 0.84 | 0.02 | 3.75 |
| 10. | How many penguins are there in Spain? ^#^ | Quantity | penguins | 43.30 | 70.57 | 0.00 | 400.00 |
| 11. | What is the maximum speed of a cheetah? | Speed | km/h | 118.04 | 61.11 | 10.00 | 435.00 |
| 12. | How many proper planets are there in our solar system? ^#^ | Quantity | planets | 9.48 | 1.80 | 6.00 | 15.00 |
| 13. | How heavy is an ironing board? ^#^ | Weight | kg | 4.45 | 2.79 | 0.23 | 12.70 |
| 14. | What is the length of an average men’s mountain bike (from tyre to tyre)? | Length | m | 1.56 | 0.45 | 0.30 | 3.66 |
| 15. | How long is the average shoelace?* | Length | cm | 54.34 | 27.59 | 0.60 | 203.00 |
| 16. | What is the population of Great Britain? ^#^ | Quantity | million | 46.04 | 20.81 | 1.00 | 100.00 |
| 17. | How tall is the average person in your country?* | Length | m | 1.72 | 0.05 | 1.52 | 1.92 |
| 18. | How many keys are there on a standard computer keyboard? | Quantity | keys | 51.56 | 15.17 | 25.00 | 126.00 |
| 19. | How many eyelashes are there on one eyelid?* | Quantity | eyelashes | 41.80 | 23.93 | 8.00 | 100.00 |
| 20. | How heavy is an average bar of soap? ^#^ | Weight | g | 135.78 | 83.40 | 2.00 | 400.00 |
| 21. | How many seats are there in a long distance single-decker coach? ^#^ | Quantity | seats | 48.18 | 17.94 | 14.00 | 212.00 |
| 22. | What is the height of the Eiffel Tower? ^#^ | Length | m | 182.23 | 100.64 | 6.00 | 500.00 |
| 23. | How heavy is a World Cup football? ^#^ | Weight | kg | 1.01 | 0.78 | 0.01 | 3.18 |
| 24. | What is the average walking speed of the typical healthy adult man? | Speed | km/h | 6.11 | 4.09 | 0.81 | 40.23 |
| 25. | How long is the average tie? | Length | m | 0.86 | 0.35 | 0.11 | 2.74 |
| 26. | What is the fastest tennis serve? | Speed | km/h | 166.62 | 74.62 | 2.00 | 483.00 |
| 27. | What is the area (length and breadth) of a beach towel? ^#^ | Area | m^2^ | 1.41 | 0.77 | 0.03 | 3.65 |
| 28. | How many keys are there on a standard piano? | Quantity | keys | 66.18 | 32.35 | 4.00 | 200.00 |
| 29. | How many camels are there in Holland? ^#^ | Quantity | camels | 9.37 | 12.54 | 0.00 | 50.00 |
| 30. | What is the age of the oldest person in your country? | Quantity | age | 116.00 | 6.76 | 102.00 | 140.00 |
| 31. | How heavy is a standard house brick? ^#^ | Weight | kg | 1.63 | 0.90 | 0.01 | 4.08 |
| 32. | What is the average area (length and breadth) of a typical hinged door? ^#^ | Area | m^2^ | 1.86 | 0.39 | 0.91 | 2.81 |
| 33. | What is the length of an average man’s spine? | Length | cm | 87.90 | 22.26 | 18.00 | 183.00 |
| 34. | What is the length of a Boeing 747? ^#^ | Length | m | 76.50 | 52.13 | 6.00 | 250.00 |
| 35. | What is the maximum speed of a cyclist? | Speed | km/h | 57.85 | 32.40 | 6.00 | 241.00 |
| 36. | How many strings are there on a harp? | Quantity | strings | 41.22 | 34.05 | 4.00 | 267.00 |
| 37. | What is the maximum speed of a Formula 1 car? | Speed | km/h | 346.88 | 89.73 | 84.00 | 805.00 |
| 38. | How tall is the average 6 year old boy?* | Length | m | 1.05 | 0.19 | 0.61 | 2.50 |

^#^ More than 10% participants scored 3 points.

* Excluded from final CET versions.
