# Supplementary material for: Bringing the Cognitive Estimation Task into the 21st Century: Normative Data on Two New Parallel Forms
Source: PLoS One. 2014 Mar 26;9(3):e92554. doi: 10.1371/journal.pone.0092554 (PMC3966793; doi:10.1371/journal.pone.0092554)
Supplement: Table S2 — Percentiles for individual items on the CET. (DOCX) [file pone.0092554.s002.docx]

|  | Percentiles | | | | | |
| --- | --- | --- | --- | --- | --- | --- |
| Item | 5^th^ | 10^th^ | 20^th^ | 80^th^ | 90^th^ | 95^th^ |
| 1 | 1.00 | 1.00 | 1.52 | 6.46 | 6.50 | 9.00 |
| 2 | 145.00 | 161.00 | 193.00 | 290.00 | 322.00 | 334.00 |
| 3 | 0.74 | 1.11 | 1.62 | 3.73 | 4.61 | 5.94 |
| 4 | 23.00 | 25.00 | 30.00 | 53.00 | 60.00 | 61.00 |
| 5 | 20.28 | 32.19 | 40.23 | 72.42 | 91.40 | 96.56 |
| 6 | 3.22 | 4.83 | 6.44 | 14.16 | 16.09 | 16.09 |
| 7 | 8.00 | 8.00 | 10.00 | 16.00 | 16.00 | 19.50 |
| 8 | 10.00 | 10.80 | 15.00 | 20.00 | 23.00 | 25.00 |
| 9 | 0.23 | 0.28 | 0.45 | 1.92 | 2.27 | 3.00 |
| 10 | 0.00 | 0.00 | 0.00 | 100.00 | 150.00 | 200.00 |
| 11 | 49.25 | 64.00 | 80.00 | 138.00 | 189.00 | 269.75 |
| 12 | 7.00 | 8.00 | 8.00 | 11.00 | 12.00 | 13.00 |
| 13 | 1.00 | 1.50 | 2.00 | 6.80 | 9.07 | 10.00 |
| 14 | 0.91 | 1.02 | 1.22 | 1.83 | 2.00 | 2.23 |
| 15 | 18.40 | 25.40 | 30.50 | 76.20 | 94.46 | 100.00 |
| 16 | 6.95 | 11.80 | 20.00 | 60.00 | 63.10 | 65.10 |
| 17 | 1.65 | 1.68 | 1.68 | 1.76 | 1.78 | 1.80 |
| 18 | 35.00 | 40.00 | 40.00 | 60.00 | 70.00 | 80.00 |
| 19 | 15.00 | 18.00 | 20.00 | 60.00 | 80.00 | 100.00 |
| 20 | 25.00 | 40.00 | 57.00 | 200.00 | 227.00 | 290.00 |
| 21 | 30.00 | 32.00 | 35.00 | 56.00 | 64.00 | 80.00 |
| 22 | 32.00 | 61.00 | 91.00 | 290.00 | 305.00 | 347.50 |
| 23 | 0.11 | 0.20 | 0.33 | 1.50 | 2.00 | 2.76 |
| 24 | 3.21 | 3.22 | 4.02 | 8.04 | 8.05 | 9.66 |
| 25 | 0.30 | 0.45 | 0.61 | 1.07 | 1.22 | 1.36 |
| 26 | 33.20 | 48.00 | 98.80 | 229.00 | 243.80 | 274.00 |
| 27 | 0.37 | 0.50 | 0.74 | 2.00 | 2.57 | 3.00 |
| 28 | 30.00 | 32.00 | 40.00 | 88.00 | 100.00 | 120.00 |
| 29 | 0.00 | 0.00 | 0.00 | 20.00 | 25.80 | 34.80 |
| 30 | 106.00 | 108.50 | 110.00 | 121.00 | 125.00 | 129.75 |
| 31 | 0.46 | 0.68 | 0.91 | 2.27 | 3.00 | 3.18 |
| 32 | 1.11 | 1.37 | 1.55 | 2.11 | 2.42 | 2.58 |
| 33 | 50.00 | 61.00 | 75.00 | 100.00 | 111.00 | 122.00 |
| 34 | 18.00 | 25.00 | 30.00 | 120.00 | 152.00 | 200.00 |
| 35 | 16.00 | 19.00 | 32.00 | 80.00 | 97.00 | 113.00 |
| 36 | 8.20 | 12.00 | 17.00 | 60.00 | 80.00 | 98.00 |
| 37 | 209.25 | 240.50 | 290.00 | 402.00 | 451.00 | 483.00 |
| 38 | 0.76 | 0.87 | 0.91 | 1.21 | 1.22 | 1.24 |

* p>.05
