# Supplementary material for: Bringing the Cognitive Estimation Task into the 21st Century: Normative Data on Two New Parallel Forms
Source: PLoS One. 2014 Mar 26;9(3):e92554. doi: 10.1371/journal.pone.0092554 (PMC3966793; doi:10.1371/journal.pone.0092554)
Supplement: Table S3 — Internal consistency for the 24 CET items. (DOCX) [file pone.0092554.s003.docx]

| Item | Corrected Item-Total Correlation | Alpha if Item Deleted |
| --- | --- | --- |
| 1 | .24 | .61 |
| 2 | .20 | .62 |
| 3 | .26 | .61 |
| 4 | .34 | .60 |
| 5 | .25 | .62 |
| 6 | .16 | .62 |
| 7 | .21 | .62 |
| 8 | .15 | .63 |
| 11 | .32 | .60 |
| 14 | .14 | .63 |
| 15 | .25 | .61 |
| 17 | .32 | .60 |
| 18 | .27 | .61 |
| 19 | .24 | .62 |
| 24 | .21 | .62 |
| 25 | .18 | .62 |
| 26 | .30 | .61 |
| 28 | .15 | .63 |
| 30 | .24 | .61 |
| 33 | .20 | .62 |
| 35 | .26 | .61 |
| 36 | .34 | .60 |
| 37 | .25 | .62 |
| 38 | .16 | .62 |
