# Supplementary material for: Bringing the Cognitive Estimation Task into the 21st Century: Normative Data on Two New Parallel Forms
Source: PLoS One. 2014 Mar 26;9(3):e92554. doi: 10.1371/journal.pone.0092554 (PMC3966793; doi:10.1371/journal.pone.0092554)
Supplement: Table S4 — Means with standard deviations in parentheses per age, gender and education group for 184 participants performing version A of the CET. (DOCX) [file pone.0092554.s004.docx]

|  |  | Age (years) | | | | | |
| --- | --- | --- | --- | --- | --- | --- | --- |
| Education (years) | Gender | 18-29 | 30-39 | 40-49 | 50-59 | 60-69 | 70-79 |
| 9-11 | M | 6.00 | 6.67 | 7.20 | 3.00 | 2.50 | 8.00 |
|  |  | (1.41) | (4.04) | (1.30) | (4.36) | (1.76) | (8.49) |
|  | F | 7.80 | - | 5.00 | 4.33 | 6.83 | 9.80 |
|  |  | (3.42) | - | (4.58) | (4.16) | (2.14) | (8.29) |
| 12-15 | M | 3.89 | 6.25 | 6.33 | 3.33 | 3.67 | 5.00 |
|  |  | (1.76) | (2.63) | (3.51) | (1.75) | (4.72) | (2.58) |
|  | F | 6.60 | 7.75 | 5.43 | 7.00 | 3.80 | 3.20 |
|  |  | (3.05) | (2.06) | (3.46) | (4.00) | (2.95) | (2.49) |
| 16-22 | M | 3.14 | 4.40 | 3.60 | 1.20 | 5.67 | 2.67 |
|  |  | (1.95) | (4.28) | (2.30) | (1.30) | (4.04) | (3.79) |
|  | F | 5.88 | 5.64 | 6.00 | 4.33 | 2.67 | 5.20 |
|  |  | (3.40) | (2.58) | (3.22) | (2.90) | (2.07) | (4.09) |

M = Male; F = Female
