# Supplementary material for: Bringing the Cognitive Estimation Task into the 21st Century: Normative Data on Two New Parallel Forms
Source: PLoS One. 2014 Mar 26;9(3):e92554. doi: 10.1371/journal.pone.0092554 (PMC3966793; doi:10.1371/journal.pone.0092554)
Supplement: Table S5 — Means with standard deviations in parentheses per age, gender and education group for 184 participants performing version B of the CET. (DOCX) [file pone.0092554.s005.docx]

|  |  | Age (years) | | | | | |
| --- | --- | --- | --- | --- | --- | --- | --- |
| Education (years) | Gender | 18-29 | 30-39 | 40-49 | 50-59 | 60-69 | 70-79 |
| 9-11 | M | 8.50 | 5.33 | 3.20 | 4.67 | 3.67 | 5.00 |
|  |  | (3.54) | (3.79) | (2.28) | (1.53) | (3.33) | (5.66) |
|  | F | 9.00 | - | 7.67 | 10.33 | 7.33 | 10.60 |
|  |  | (5.29) | - | (6.43) | (4.93) | (4.50) | (7.77) |
| 12-15 | M | 5.89 | 5.25 | 4.67 | 1.67 | 4.67 | 1.50 |
|  |  | (3.22) | (3.86) | (4.51) | (1.75) | (1.86) | (1.29) |
|  | F | 9.60 | 9.25 | 5.57 | 7.57 | 5.80 | 5.80 |
|  |  | (3.85) | (6.55) | (2.44) | (2.57) | (2.17) | (4.55) |
| 16-22 | M | 5.71 | 2.80 | 3.20 | 3.20 | 6.33 | 1.33 |
|  |  | (1.50) | (2.39) | (2.59) | (2.49) | (4.73) | (0.58) |
|  | F | 5.25 | 5.36 | 4.83 | 4.67 | 4.67 | 3.20 |
|  |  | (4.23) | (3.23) | (2.14) | (3.89) | (1.21) | (1.92) |

M = Male; F = Female
