# Supplementary material for: Bringing the Cognitive Estimation Task into the 21st Century: Normative Data on Two New Parallel Forms
Source: PLoS One. 2014 Mar 26;9(3):e92554. doi: 10.1371/journal.pone.0092554 (PMC3966793; doi:10.1371/journal.pone.0092554)
Supplement: Table S7 — The correction grid with the points to add or subtract from the raw scores to obtain adjusted scores for version B of the CET. For the combinations not reported, the corrections that should be applied to the raw CET scores to achieve adjusted scores are below the table. (DOCX) [file pone.0092554.s007.docx]

|  |  | Education (years) | | | | | | | | | |
| --- | --- | --- | --- | --- | --- | --- | --- | --- | --- | --- | --- |
| Age | Gender | 9 | 10 | 11 | 12 | 13 | 14 | 15 | 16 | 17 | 18 |
| 20 | M | 3 | 3 | 2 | 2 | 2 | 1 | 1 | 0 | 0 | 0 |
|  | F | 6 | 5 | 5 | 5 | 4 | 4 | 3 | 3 | 3 | 2 |
| 25 | M | 3 | 3 | 2 | 2 | 1 | 1 | 1 | 0 | 0 | -1 |
|  | F | 6 | 5 | 5 | 4 | 4 | 4 | 3 | 3 | 2 | 2 |
| 30 | M | 3 | 2 | 2 | 2 | 1 | 1 | 0 | 0 | 0 | -1 |
|  | F | 5 | 5 | 5 | 4 | 4 | 3 | 3 | 3 | 2 | 2 |
| 35 | M | 3 | 2 | 2 | 1 | 1 | 1 | 0 | 0 | -1 | -1 |
|  | F | 5 | 5 | 4 | 4 | 4 | 3 | 3 | 2 | 2 | 2 |
| 40 | M | 2 | 2 | 2 | 1 | 1 | 0 | 0 | 0 | -1 | -1 |
|  | F | 5 | 5 | 4 | 4 | 3 | 3 | 3 | 2 | 2 | 1 |
| 45 | M | 2 | 2 | 1 | 1 | 1 | 0 | 0 | -1 | -1 | -1 |
|  | F | 5 | 4 | 4 | 4 | 3 | 3 | 2 | 2 | 2 | 1 |
| 50 | M | 2 | 2 | 1 | 1 | 0 | 0 | 0 | -1 | -1 | -2 |
|  | F | 5 | 4 | 4 | 3 | 3 | 3 | 2 | 2 | 1 | 1 |
| 55 | M | 2 | 1 | 1 | 1 | 0 | 0 | -1 | -1 | -1 | -2 |
|  | F | 4 | 4 | 4 | 3 | 3 | 2 | 2 | 2 | 1 | 1 |
| 60 | M | 2 | 1 | 1 | 0 | 0 | 0 | -1 | -1 | -2 | -2 |
|  | F | 4 | 4 | 3 | 3 | 3 | 2 | 2 | 1 | 1 | 1 |
| 65 | M | 1 | 1 | 1 | 0 | 0 | -1 | -1 | -1 | -2 | -2 |
|  | F | 4 | 4 | 3 | 3 | 2 | 2 | 2 | 1 | 1 | 0 |
| 70 | M | 1 | 1 | 0 | 0 | 0 | -1 | -1 | -2 | -2 | -2 |
|  | F | 4 | 3 | 3 | 3 | 2 | 2 | 1 | 1 | 1 | 0 |
| 75 | M | 1 | 1 | 0 | 0 | -1 | -1 | -1 | -2 | -2 | -3 |
|  | F | 4 | 3 | 3 | 2 | 2 | 2 | 1 | 1 | 0 | 0 |
| 80 | M | 1 | 0 | 0 | 0 | -1 | -1 | -2 | -2 | -2 | -3 |
|  | F | 3 | 3 | 3 | 2 | 2 | 1 | 1 | 1 | 0 | 0 |

Version B (Male):

K = [-0.04 x (age – 48.07)] – [0.40 x (education – 14.33)]

Version B (Female):

K = [-0.04 x (age – 48.07)] – [0.40 x (education – 14.33)] + 2.54
